# FACILITATING CAREGIVER HEALTH AND WELLNESS: AGE-FRIENDLY HEALTH SYSTEM CARING FOR CAREGIVERS (AFHS-C4C)

**DOI:** 10.1093/geroni/igac059.549

**Published:** 2022-12-20

**Authors:** Robyn Golden, Diane Mariani, Leslie Pelton, Teresa Moro, Ellen Carbonell

**Affiliations:** Rush University Medical Center, Chicago, Illinois, United States; Rush University Medical Center, Chicago, Illinois, United States; Institute for Healthcare Improvement, Boston, Massachusetts, United States; Rush University Medical Center, Chicago, Illinois, United States; Rush University Medical Center, Chicago, Illinois, United States

## Abstract

Family caregivers of older adults are vital partners in health care. The health care system is uniquely positioned to connect with caregivers and help address their own needs. The Age-Friendly Health System Caring for Caregivers (AFHS-C4C) model focuses on achieving health system change by identifying and incorporating caregivers into standard health care practices while assisting caregivers with their own health and well-being. Created at Rush University Medical Center, this model was tested in six AFHS. At Rush, caregivers (n=322) were mostly female (74%) and African American (38%) or white (37%). Between baseline and follow-up, statistically significant (p<.05) decreases were observed in caregiver burden (BSFC: 18.51 to 13.82), depression (PHQ-9: 8.40 to 4.09), and anxiety (GAD-7: 7.73 to 5.15). Application of this program at additional sites has yielded important lessons learned about engagement, training needs, and sustainability. These lessons will be discussed in the context of scaling and spreading interventions nationally.

